# Evidence Supporting a Role for Mammalian Chitinases in Efficacy of Caspofungin against Experimental Aspergillosis in Immunocompromised Rats

**DOI:** 10.1371/journal.pone.0075848

**Published:** 2013-10-14

**Authors:** Patricia E. B. Verwer, Marian T. ten Kate, Franco H. Falcone, Shaun Morroll, Henri A. Verbrugh, Irma A. J. M. Bakker-Woudenberg, Wendy W. J. van de Sande

**Affiliations:** 1 Department of Medical Microbiology and Infectious Diseases, Erasmus University Medical Centre Rotterdam, Rotterdam, The Netherlands; 2 Division of Molecular and Cellular Science, The School of Pharmacy, University of Nottingham, Nottingham, United Kingdom; University of Wisconsin - Madison, United States of America

## Abstract

**Objectives:**

Caspofungin, currently used as salvage therapy for invasive pulmonary aspergillosis (IPA), strangely only causes morphological changes in fungal growth *in vitro* but does not inhibit the growth. *In vivo* it has good efficacy. Therefore the question arises how this *in vivo* activity is reached. Caspofungin is known to increase the amount of chitin in the fungal cell wall. Mammals produce two chitinases, chitotriosidase and AMCase, which can hydrolyse chitin. We hypothesized that the mammalian chitinases play a role in the *in vivo* efficacy of caspofungin.

**Methods:**

In order to determine the role of chitotriosidase and AMCase in IPA, both chitinases were measured in rats which did or did not receive caspofungin treatment. In order to understand the role of each chitinase in the breakdown of the caspofungin-exposed cells, we also exposed caspofungin treated fungi to recombinant enzymes *in vitro*.

**Results:**

IPA in immunocompromised rats caused a dramatic increase in chitinase activity. This increase in chitinase activity was still noted when rats were treated with caspofungin. *In vitro*, it was demonstrated that the action of both chitinases were needed to lyse the fungal cell wall upon caspofungin exposure.

**Conclusion:**

Caspofungin seemed to alter the cell wall in such a way that the two chitinases, when combined, could lyse the fungal cell wall and assisted in clearing the fungal pathogen. We also found that both chitinases combined had a direct effect on the fungus *in vitro*.

## Introduction

*Aspergillus fumigatus* is a ubiquitous saprophytic fungus, producing conidia that are inhaled daily by humans. Usually inhalation of these spores causes no problem; however, *A. fumigatus* can cause a broad range of diseases in hosts with underlying conditions. Patients with inflammatory conditions, such as asthma and cystic fibrosis, can develop allergic bronchopulmonary aspergillosis (ABPA) [1], [2]. Patients with prolonged neutropenia are at risk to develop invasive pulmonary aspergillosis (IPA) [3]. In neutropenic patients, IPA may be characterized by necrotizing pneumonia or hemorrhagic infarctions. Due to the progressive character of IPA, the morbidity and mortality is high. The response to treatment is limited, despite the application of several antifungal agents with different mechanisms of action.

The current first choice antifungal agent for IPA is voriconazole, with amphotericin B as the alternative therapy [3]. Caspofungin is available as salvage therapy, in case of refractory disease or intolerance of voriconazole or amphotericin B by the patient. Strikingly, *in vitro* caspofungin has only a mediocre activity against *A. fumigatus*. It does not have fungicidal or fungistatic activity. Only at a very high concentration growth inhibition is noted. However, in *in vivo* animal models caspofungin seems to be very potent. In our transiently neutropenic rat model with unilateral invasive pulmonary aspergillosis, the human equivalent dosage of caspofungin results in 100% efficacy [4]. Therefore the question arises what the reason is for this discrepancy between *in vivo* and *in vitro* results.

One hypothesis would be that the immune system of the host plays a role. Caspofungin acts by inhibiting β-glucan synthase thereby decreasing the β-glucan contents of the fungal cell wall. To restore the stability of the cell wall, the fungus reacts by increasing its other major cell wall component, chitin, as demonstrated *in vitro* [5], [6].

Chitin can be cleaved by chitinases, which belong to family 18 of glycosyl hydrolases and are produced in cooperation with the immune system. Chitinases are classified into endochitinases and exochitinases. Exochitinases act at the non-reducing ends of chitin with the release of successive diacetyl chitobiose units. In contrast, endochitinases randomly cleave at internal points in the chitin chain [7], [8]. Mammals are known to produce two types of chitinases: chitotriosidase and acidic mammalian chitinase (AMCase) [9]. Chitotriosidase is produced by macrophages and polymorphonuclear neutrophils [10], [11] and can be found in the lungs of mammals [12], [13] as well as in lacrimal glands [14]. AMCase is an exochitinase produced by macrophages and epithelial cells [15] and is found mainly in the gastro-intestinal tract of mammals to digest nutritional chitin, though it was also found in the lung at low concentrations [10]. The exact role of chitinases remains to be clarified. However, an important role for chitinases in allergic diseases has been suggested [16], [17].

In the past it was already demonstrated by Overdijk *et al.* that chitinase activity was increased in plasma isolated from guinea pigs with a systemic *A. fumigatus* infection. Furthermore, high chitinase levels were detected in the spleen, followed by lungs and kidneys [18], [19]. However, these results did not reveal whether the chitinase activity was due to chitotriosidase or AMCase activity, or a combination of both since at that time, AMCase had not been discovered as a chitinase yet [10].

Summarizing the above, we can state that the chitin content in the *A. fumigatus* cell wall is increased upon exposure to caspofungin and mammalian chitinases are induced during invasive aspergillosis. We therefore hypothesized that either chitotriosidase, AMCase or both play a role in the clearing of *A. fumigatus* from the lung when treated with caspofungin. In order to test this hypothesis, we first determined which of these two chitinases was induced upon *A. fumigatus* conidia in both immunocompetent rats, clearing the conidia, and in immunocompromised rats, suffering from invasive pulmonary aspergillosis. In these experiments we determined if chitinases could be induced in the first place during the neutropenic state. Next, we determined if both chitotriosidase and AMCase were more extensively expressed during caspofungin treatment. Afterwards we investigated how these two chitinases and caspofungin interact and what the combined effect is on the *A. fumigatus* hyphae *in vitro*.

## Materials and Methods

### Experimental animal model

The rat model of invasive pulmonary aspergillosis (IPA) in immunocompromised rats used, was described previously [20]. Some minor changes have led to the following experimental set up.

In order to determine if *A. fumigatus* conidia induce chitinase activity, immunocompetent female albino RP rats were inoculated intratracheally with a clinical isolate of *Aspergillus fumigatus* originally isolated from a hemato-oncological patient with IPA. Left-sided pulmonary inoculation was established by intubation of the left main bronchus, while the rats were under general anaesthesia. A cannula was passed through the tube and the left lung was inoculated with 20 µl phosphate buffered saline (pbs) containing 6×10^4^ conidia of *A. fumigatus*. Rats were sacrificed at day 1, 3 and 6 after fungal inoculation of the rats to determine the chitotriosidase and AMCase activity both enzymatically and immunohistochemically. For this, blood samples were taken by puncture of the orbital plexus and rats were sacrificed by CO_2_ exposure. The left lung was removed and either stored at −80°C until analysis, or fixated in formalin for immunohistochemistry. Serum was also stored at −80°C until analysis. The groups consisted of a minimum of 4 rats. Infected organs and blood from rats found dead were always cultured to exclude bacterial superinfections. Fungal load was assessed by determination of serum galactomannan index (GM-index), using the commercially available Platelia *Aspergillus* EIA Platelia *Aspergillus* system of BioRad (Marnes-la-Coquette, France).

In order to determine if *A. fumigatus* conidia induce chitinase activity in neutropenic female albino RP rats developing IPA, transient neutropenia was induced by intraperitoneally (i.p.) administered cyclophosphamide (Endoxan, Baxter, Utrecht, The Netherlands) in doses of 75, 60, 50 and 40 mg/kg bodyweight at 5 and 1 days before fungal inoculation, and at 3 and 7 days after fungal inoculation, respectively. Whereas the normal leukocyte counts in our rats is 5.8×10^9^/L, the leukocyte counts decreased following cyclophosphamide treatment and were 6.5×10^7^/L on the day of fungal inoculation and 6.4×10^7^/L on day 5 and day 9 after inoculation. After the last dosage of cyclophosphamide, leukocyte counts rose to 2.6×10^9^/L on day 13 and to 6.0×10^9^/L on day 21 after infection. Granulocyte counts decreased from 2×10^8^/L before cyclophosphamide was given (normal counts in our rats) to 2×10^4^/L from the day of fungal inoculation to day 9. After the final dosage of cyclophosphamide, granulocyte counts increased again to 1.2×10^7^/L and 1.2×10^9^/L on day 13 and 21 after infection, respectively.

To prevent bacterial superinfections, rats were given ciprofloxacin (500 mg/L) and colistin (100 mg/L) in their drinking water. Furthermore, rats were given teicoplanin intramuscularly (i.m.) in doses of 30 mg/kg on days 5 and 1 pre- inoculation, and 15 mg/kg on days 1, 3, 6, 8 and 10 post-inoculation. Immunocompetent rats were injected with saline i.p. instead of cyclophosphamide.

Left-sided pulmonary infection was established, by intubation as described for the immunocompetent rats. Again, rats were sacrificed at day 1, 3 and 6 after fungal inoculation of the rats to determine the chitotriosidase and AMCase activity both enzymatically as immunohistochemically as described for the immunocompetent rats.

Since rats were sacrificed at these predetermined time points, death of rats was no primary endpoint. Rats were monitored according to a discomfort scale by the researchers several times a day, during the entire experiment. In order to limit suffering, rats were euthanized in case of high discomfort, shown by e.g. increased breathing exercise, increased respiratory rates and altered behaviour, like decreased movements and unkempt appearance (dull haircoat). The experimental protocols adhered to the rules specified in the Dutch Animal Experimentation Act (1977) and the *Guidelines on the Protection of Experimental Animals* published by the Council of the EC (7a). The present protocols were approved by the Institutional Animal Care and Use Committee of the Erasmus MC Rotterdam.

### Antifungal treatment

Caspofungin (Merck & Company, Rahway, NJ, USA) was diluted in saline and administered intraperitoneally once daily in a dose of 4 mg/kg/day. Treatment was started at 24 h (early stage IPA) or at 72 h (late stage IPA) after fungal inoculation. Treatment was continued for six days.

### Recombinant expression of rat AMCase

Recombinant rat AMCase was generated using the pMIB insect cell expression system by Invitrogen as previously described for human chitotriosidase [14]. The primers had *SphI* (forward primer) and *Xba*1 (reverse primer) restriction sites at the 5′ ends that facilitated the in-frame cloning into pMIB/V5-His (Invitrogen). The primer sequences were as follows: RnCHIA-F 5′-GCCCGGGCATGCATtacaatctggtatgctacttcac-3′ and RnCHIA-R 5′- GCCCGGTCTAGAtggccagttgcagcaattacagctg-3′ (restriction sites underlined). The PCR reactions for generation of full length AMCase for expression cloning were conducted using *Pfu* DNA polymerase (Stratagene) following the manufacturer's instructions for a 50 µl reaction using cDNA obtained from rat lung tissue. The final sequence of the recombinant plasmid was confirmed by DNA sequencing. The activity of the recombinant rat AMCase produced here and the purchased recombinant chitotriosidase were determined with the chitinase assays as described below.

### Chitinase assay

Chitinase activity was determined in homogenized lung tissue. Activity of 4-Methylumbelliferyl N,N′-diacetyl-β-D-chitobioside (chitobiosidase activity, corresponding with AMCase activity) and 4-Methylumbelliferyl β-D-N,N′,N″-triacetylchitotriose (endochitinase activity, corresponding with CHIT1 activity) were determined using the commercial fluorimetric Chitinase Assay Kit (Sigma-Aldrich Chemie GmbH, Steinheim, Germany). Chitinase activity in each sample was measured in duplicate. Chitinase activity was expressed in arbitrary units (a.u.) and median chitinase activity levels were compared for different groups of rats. Chitinase activity from *Trichoderma viride* (control enzyme from the Chitinase Assay Kit described above) was used as a positive control. A positive and a negative control were used in each run, in order to validate the experiment.

Chitotriosidase and AMCase activity were measured in homogenized lungs. It was confirmed that all chitinase activity was indeed of host origin as follows: *A. fumigatus* conidia were cultured for 48 hours at 37°C in Sabouraud's broth or RPMI, with and without caspofungin 1 mg/L. The broth was filtered after 48 h and chitinase activity in the broth was measured in triplicate. Activity of chitotriosidase and AMCase was measured with the commercial fluorimetric chitinase assay kit and was found to be <5 a.u. in all samples tested. We thus concluded that the chitinases measured in the lung tissue were indeed of host origin and not of fungal origin.

### Immunohistochemistry of lungs and *in vitro* cultures

Lungs were fixed in formalin, embedded in paraffin and processed for immunohistochemical evaluation. First, lungs were deparaffinised in xylene, then rehydrated in decreasing concentrations of ethanol. Endogenous peroxidase was blocked in methanol with 0.3% H_2_O_2_ and non-specific binding sites were blocked with rabbit or goat serum. Subsequently, coupes were incubated overnight with rabbit polyclonal antibody directed against chitotriosidase (H-66, 1∶75, Santa Cruz Biotechnology, Santa Cruz, USA) or with goat polyclonal antibody directed against AMCase (Y-14, 1∶50, both Santa Cruz Biotechnology, Santa Cruz, USA). As a control we used a goat polyclonal IgG antibody directed against swine IgM (A100-100A, 1∶50, Bethyl Laboratories, Montgomery, USA). From the VectaStain® Elite ABC kit (Vector Laboratories Burlingame, CA, USA), anti-rabbit IgG or anti-goat IgG was used as a secondary antibody and the coupes were developed using the protocol from the kit. Hematoxylin was used as counter staining. In order to ascertain that the antibodies did not react with chitinases expressed by *A. fumigatus* itself, *A. fumigatus* was grown on the same histological slides and fixated. These fixated *A. fumigatus* slides were stained according to the same protocol used for the histological slides. No staining of either chitotriosidase or AMCase was observed.

### *In vitro* binding of chitinases to fungal hyphae

*Aspergillus fumigatus* was cultured for 48 h at 37°C on Sabouraud's agar with or without 1 mg/L caspofungin, on which a cover slip had been placed. The fungus adhered to the cover slip and after 48 h the cover slips were removed and processed. Cover slips were incubated with 0.5 mg/L recombinant chitotriosidase (purchased from Sigma-Aldrich Chemie GmbH, Steinheim, Germany) or with 0.5 mg/L recombinant AMCase (recAMCase; School of Pharmacy, Nottingham, UK), both or with aquadest for 2 hours at 37°C. Afterwards, cover slips were fixed in 70% and 100% ethanol. Non-specific binding sites were blocked with rabbit or goat serum. Cover slips were further processed identical to the lung coupes; however no counter staining was used.

### Determination of *in vitro* inhibitory concentrations

Minimal inhibitory concentrations (MICs) were determined for recAMCase, recChito and chitinase from *T. viride* (see section “Chitinase assay”) according to the microdilution methods described by the CLSI [21]. Final concentrations of recAMCase were 0.031–128 mg/L. Final concentrations of recChito were 0.031–16 mg/L. Final concentrations of chitinase from *T. viride* were 0.031–16 mg/L. Minimal effective concentration (MEC) was determined for caspofungin, according to the same guidelines [21]. Final concentrations of caspofungin were 0.063–128 mg/L.

Checkerboard titrations were conducted in triplicate for caspofungin combined with recAMCase, recChito and chitinase from *T. viride* as a positive control and fractional inhibitory concentration indices (FICIs) were calculated as previously published [6]. MEC was used for caspofungin and MICs were used for chitotriosidase and AMCase. Drug interactions were classified as synergistic (FICI≤0.5), indifferent (0.5<FICI<4) or antagonistic (FICI≥4).

### Fungal cell wall assessment by fluorescent microscopy

*Aspergillus fumigatus* was cultured on a cover slip as described above. Cover slips were incubated with either recombinant chitotriosidase alone, recombinant AMCase alone, both recombinant chitinases or with aquadest as negative control for 2 hours at 37°C. Cover slips were washed in aquadest and incubated with 25 µM Calcofluor White (Molecular Probes®, Leiden, The Netherlands) for 30 minutes at 37°C in the dark. Afterwards, cover slips were washed in aquadest and placed on a microscopic slide and assessed by fluorescent microscopy.

### Statistics

Differences in chitinase activity levels between groups were analysed using the Mann-Whitney U-test (GraphPad Prism Software, San Diego, USA). A p-value of <0.05 was considered significant.

## Results

### Chitinases in immunocompetent rats inoculated with *A. fumigatus*

In order to determine if *A. fumigatus* conidia induce chitinase activity, immunocompetent rats were inoculated intratracheally with *A. fumigatus* conidia. After inhalation of the inoculum, all rats remained asymptomatic and none of the rats developed invasive pulmonary aspergillosis (IPA), their serum galactomannan indices remained <0.2 and no hyphae were observed in Grocott-stained lungs on day 1, day 3 and day 6. Compared to the situation before inoculation, both chitotriosidase and AMCase activity in the lung increased on day 1 after inoculation, from 25 to 119 arbitrary units (a.u.)(chitotriosidase; p = 0.011) and from 17 to 60 a.u. (AMCase; p = 0.006). After the initial increase in chitinase activity on day 1, enzyme activity of both chitinases remained elevated on day 3 and day 6 but did not increase further when compared to day 1 (p>0.05; for all chitotriosidase and AMCase activities see Table S1). Thus, exposure of the rats to conidia resulted in increased activity of both chitotriosidase and AMCase for several days. However, the data obtained with immunohistochemistry suggested that inoculation with conidia did not result in increased presence of chitotriosidase and AMCase in the lung.

### Chitinases in immunocompromised rats with IPA

**Figure 1:**
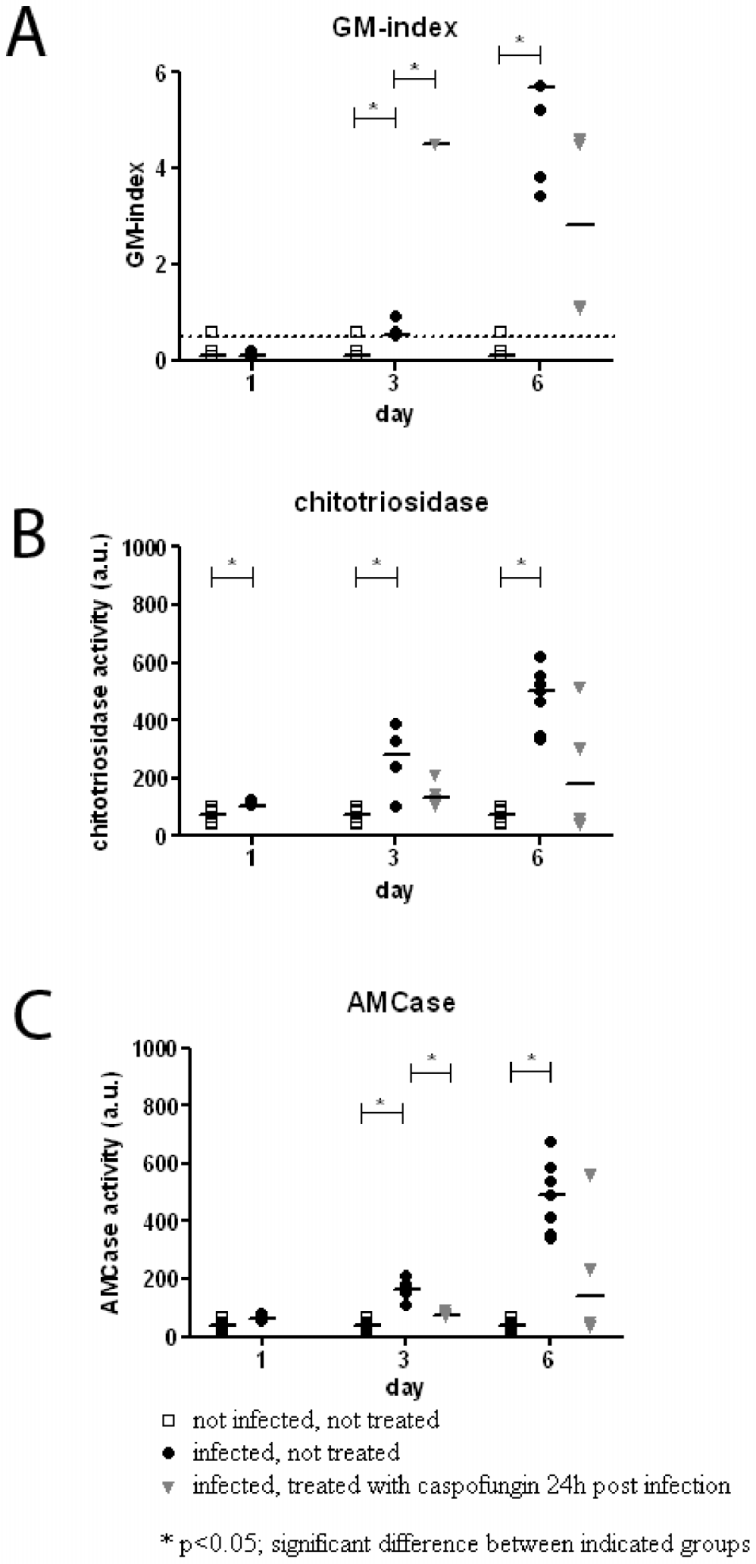
Chitinase activity and fungal load in immunocompromised rats inoculated with *A. fumigatus* conidia. Open squares: uninfected untreated rats; filled circles: infected untreated rats; grey triangles: infected rats, treated with caspofungin at 24 h post infection. Data are means of duplicates. Bars represent medians. For each group n≥4. A. Galactomannan (GM)-index, measured by Platelia assay. According to the manufacturer's manual, GM-index of <0,5 is considered negative; B, chitotriosidase activity, expressed in arbitrary units (a.u.); C, AMCase activity, expressed in arbitrary units (a.u.). * p<0.05; significant difference between the indicated groups.

**Figure 2:**
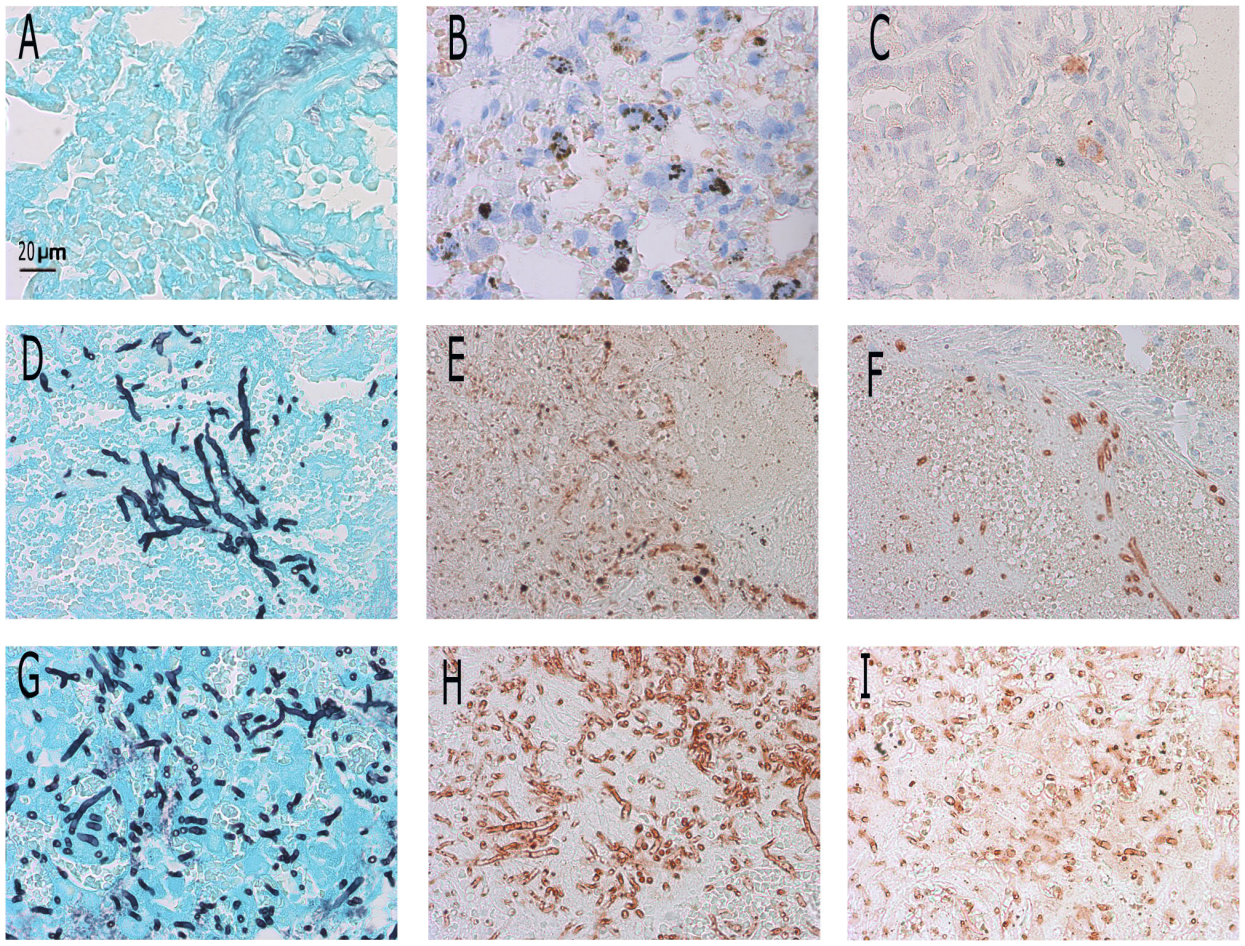
Grocott staining (A, D, G) and presence of AMCase (B, E, H) and chitotriosidase (C, F, I) in several rats. Panels A, B, and C show the lung of an uninfected rat. Panels D, E and F show the fungal focus in an infected, untreated rat. Panels G, H and I show the fungal focus in an infected, caspofungin treated rat. Original magnification ×400. All panels represent lungs on day 6 after inoculation. Slides were stained according to the described protocols. In Grocott staining (A, D, G), fungal hyphae are coloured black. Chitotriosidase- or AMCase-presenting cells are coloured red (B, C, E, F, H, I). In uninfected rats, normal morphology can be found in the lungs (A, B, C). In infected rats, normal morphology of alveoli is lost (D, E, F). Grocott staining shows many hyphae (D). An inflammatory response is found around the fungal focus, where chitotriosidase and AMCase are increasingly present (red zones) compared to an uninfected rat (E, F). After treatment with caspofungin, Grocott staining shows fungal material in all infected rats (G). AMCase bound fungal hyphae after treatment with caspofungin (H) and thus hyphae became visible. After treatment with caspofungin, chitotriosidase seemed to also bind the fungal cell wall and locate inside hyphal cells (I).

To determine if chitinases were also induced during the neutropenic state, we investigated chitinase activity in immunocompromised rats. Neutrophil depletion was induced using cyclophosphamide as described in the experimental procedures section and immunocompromised rats were inoculated intratracheally with *A. fumigatus* conidia. As previously described, all rats developed IPA and died within ten days, if left untreated [20]. Bacterial superinfections were never found. IPA was confirmed by galactomannan indices in serum (figure 1A) and histopathology of the lung (Grocott staining; figure 2D), which showed invasive fungal disease.

Chitotriosidase and AMCase activity increased during fungal infection (Figure 1B, C).

Chitotriosidase activity increased from 75 a.u. before inoculation to 105 a.u. on day 1 (p = 0.041), to 281 a.u. on day 3 and to 501 a.u. on day 6 (both p<0.001). AMCase activity increased from 41 a.u. before inoculation to 61 a.u. on day 1 (p = 0.066), 164 a.u. on day 3 and 491 a.u. on day 6 (both p<0.001; see Figure 1B,C and Table S1 for all activities). The initial increase in chitinase activity on day 1 was similar to that in immunocompetent rats. However, immunocompromised rats developed invasive fungal disease and the chitinase activities further increased, indicating that a higher fungal content was related to both a higher chitotriosidase and AMCase activity in the lung. Both chitotriosidase and AMCase activity were host specific, since no activity was found when *A. fumigatus* culture supernatant were measured.

The histology of lungs of immunocompromised rats with IPA showed disturbed lung morphology. There was an inflammatory area around fungal foci, where the normal structure of alveoli was lost (Figure 2D, E, F). After inoculation, AMCase and chitotriosidase were increasingly expressed around the fungal focus (Figure 2E, F), which was consistent with the increased chitinase activity levels as described above. The staining of both chitinases was deemed specific for host chitinases, since no staining was observed when *A. fumigatus* was cultured on a slide and stained afterwards (see above).

### Chitinase activity in immunocompromised rats with IPA treated with caspofungin

In order to determine if chitinase expression and activity differed in rats treated with caspofungin, another group of immunocompromised rats was inoculated with *A. fumigatus*. Treatment with caspofungin was started at 24 h post fungal inoculation, representing early stage IPA. Treatment started at this early time point resulted in survival of 90% of the rats [20]. Chitotriosidase and AMCase activity initially increased after fungal inoculation, as described above. In infected caspofungin-treated rats, at day 3 chitotriosidase and AMCase levels were similar to the levels in infected untreated rats (Figure 1B, C). On day 6, a trend was observed that chitotriosidase and AMCase levels were lower in infected caspofungin-treated rats compared to infected untreated rats (Figure 1B, C), however the differences were not statistically significant (for all chitinase activities: see Table S1) The same trend was found for fungal load, in terms of GM-index (Figure 1A); GM-index was 5.7 for untreated rats and 2.8 for treated rats (p = 0.053). On day 3, GM-index was lower for untreated rats than for caspofungin-treated rats, in contrast to chitinase activity. Apparently, chitinase activity is increased before GM-index rises.

Immunohistochemistry showed a similar pattern of expression of chitotriosidase and AMCase in infected caspofungin-treated and infected untreated rats. Expression of both chitinases was highest around the fungal focus. In other relatively healthy parts of the lung, expression of chitinases was similar to that in uninfected rats. Figure 2 shows the fungal focus and the location of the AMCase and chitotriosidase expressing cells. AMCase was expressed by several cells throughout the lung, though expression was also highest around the fungal focus (Figure 2E). After treatment with caspofungin, AMCase was found to bind to the fungal cell wall (Figure 2H), though not all hyphae were stained. This was not seen in hyphae of untreated animals. In unaffected parts of the infected lung, where normal morphology was maintained, AMCase expression was comparable with that in uninfected rats (data not shown). Without caspofungin treatment, chitotriosidase was found also mainly around foci of fungal growth. No distinct cell type expressing chitotriosidase could be assigned (Figure 2F). After treatment with caspofungin, chitotriosidase was found to bind the hyphal cell wall and inside the cell (Figure 2I). Expression of both chitinases was highest in close proximity to the fungal focus, regardless of treatment. Apparently, treatment with caspofungin caused such an alteration in the fungal cell wall that AMCase was able to bind the fungal hyphae and chitotriosidase was taken up by the fungus.

In order to investigate late stage IPA, we also determined chitinase activity levels in a group of rats that received caspofungin treatment starting at 72 h post fungal inoculation (late stage IPA). GM-index and immunohistochemistry revealed increased fungal load compared to rats that were treated with caspofungin starting at 24 h post inoculation (early stage IPA). For chitinase activity, we observed levels that were not significantly different between the two groups (see Table S1).

### *In vitro* binding of recombinant chitinase to hyphae of *A. fumigatus*

As described above, we observed binding of AMCase and hyphal uptake of chitotriosidase in the lung after treatment with caspofungin. We hypothesized that treatment with caspofungin was required for binding of both chitinases, since caspofungin causes increased chitin contents in the fungal cell wall [6] and thus increases the substrate for chitinases. In order to confirm our hypothesis, we investigated *in vitro* binding of recombinant AMCase and recombinant chitotriosidase to *A. fumigatus* hyphae on slides. *A. fumigatus* was cultured on cover slips in presence or absence of caspofungin, which was followed by exposure to recombinant AMCase or recombinant chitotriosidase or both for two hours. Binding of the enzyme was detected in the same way as expression was detected in the lungs taken from rats with IPA. Additionally, we tested antifungal activity of recombinant AMCase or recombinant chitotriosidase alone and combined with caspofungin in a checkerboard titration according to the CLSI criteria.

**Figure 3:**
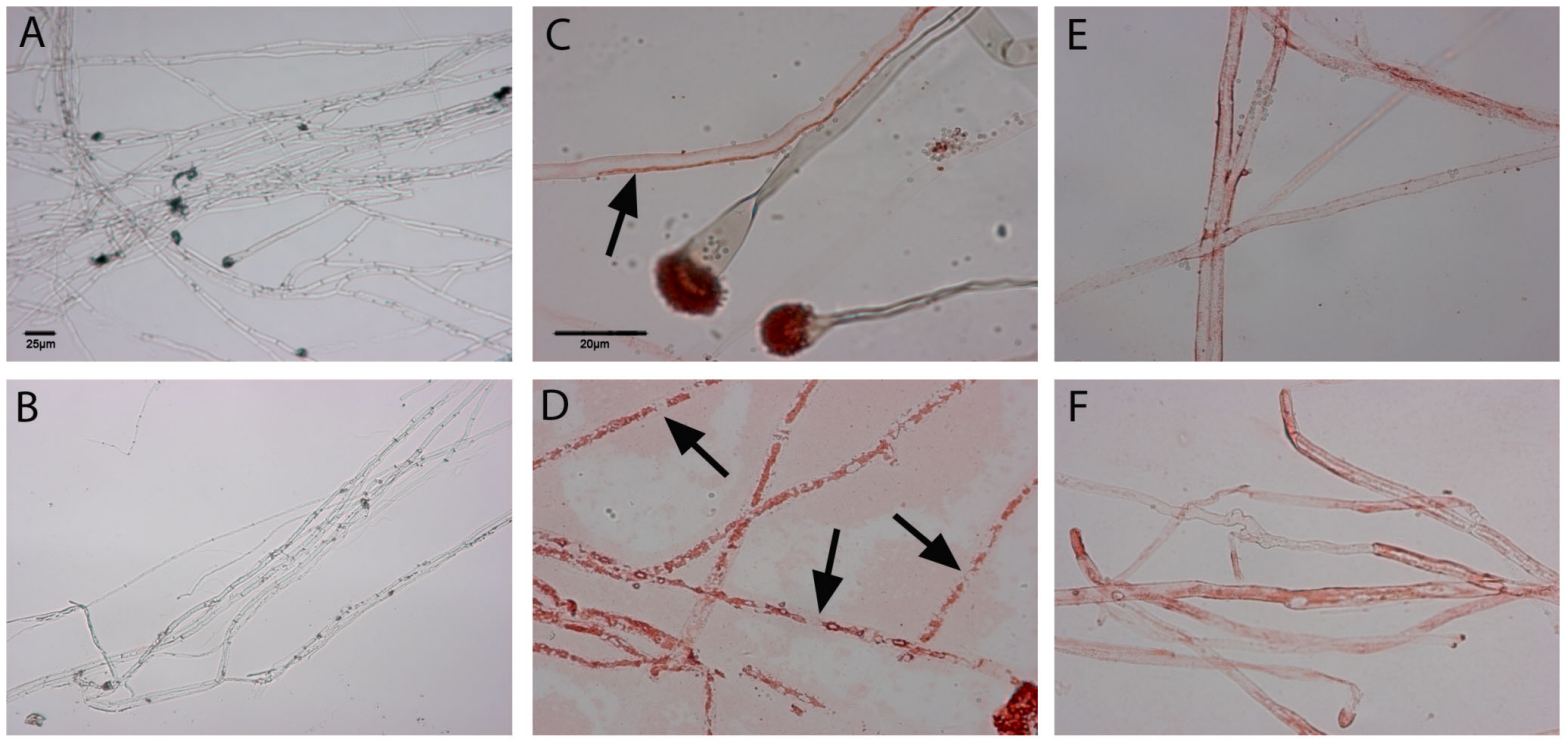
*In vitro* binding of recombinant chitinases to *A. fumigatus* hyphae. Binding of recombinant chitotriosidase (A, B). Binding of recombinant chitotriosidase when incubated in combination with recombinant AMCase (C, D) and binding of recombinant AMCase (E, F). Panels A, C and E show unexposed hyphae. Panels B, D and F show caspofungin-exposed hyphae. A, B: Contrast and brightening were slightly modified in Photoshop due to the lack of colour. C, D, E, F: Photos were not modified in Photoshop. A–B Original magnification ×100. C–F Original magnification ×400. Slides were stained according to the described protocols. Binding of either recombinant enzyme is characterized by a red colour. Recombinant chitotriosidase did not bind to unexposed hyphae (A) or to caspofungin-exposed hyphae (B). When incubated with a combination of recombinant chitotriosidase and recombinant AMCase, recombinant chitotriosidase did bind to unexposed hyphae (arrow) and to conidial heads (C) and seemed to be taken up by the fungal cells after caspofungin exposure (D). Also the cell wall seemed to be lysed at several locations (arrows). Recombinant AMCase did bind to unexposed (E) and to caspofungin-exposed hyphae (F).

Recombinant AMCase did bind similarly to fungal hyphae both in unexposed and in caspofungin-exposed fungal cells (Figure 3E, F), showing that caspofungin exposure was not needed for binding of AMCase. Susceptibility testing showed a median minimal inhibitory concentration (MIC) of recombinant AMCase of >16 mg/L. Combination of recombinant AMCase with caspofungin in a checkerboard titration showed no synergy (median fractional inhibitory concentration index [2] 2.0). Thus exposure to caspofungin and recombinant AMCase was not sufficient for antifungal activity *in vitro*.

Recombinant chitotriosidase was not taken up by *A. fumigatus* hyphae during incubation with this enzyme regardless of caspofungin exposure (Figure 3A,B), in contrast to what was seen in infected lungs. Susceptibility testing showed an MIC of recombinant chitotriosidase of >16 mg/L. Combination of recombinant chitotriosidase with caspofungin in the checkerboard titration showed again no synergy (median FICI 2.0). Thus exposure to caspofungin and recombinant chitotriosidase alone was also not sufficient for antifungal activity *in vitro*.

Strikingly the binding pattern of recombinant chitotriosidase in the infected lungs was different to the pattern found *in vitro*. *In vivo*, both chitinases were present, though only one chitinase was stained at a time. Hence we additionally investigated the binding of recombinant chitotriosidase to the fungus in presence of both recombinant enzymes *in vitro*. When hyphae were not exposed to caspofungin, chitotriosidase seemed to locate inside the hyphae and was also bound to the conidial heads (Figure 3C). When hyphae were exposed to caspofungin and to both recombinant chitinases, chitotriosidase also located inside the cell wall and strikingly, the cell wall seemed to dissolve (Figure 3D). From these experiments, we observed that recombinant chitotriosidase could only be taken up by hyphae, provided recombinant AMCase was present. Furthermore we observed that caspofungin modifies the fungal cell wall in such a way, that a combination of recombinant AMCase and recombinant chitotriosidase can lyse the fungal cell wall. The uptake of chitotriosidase and morphologic disruptions suggest chitinolytic results and thus clinical significance in the clearance of fungal material.

**Figure 4:**
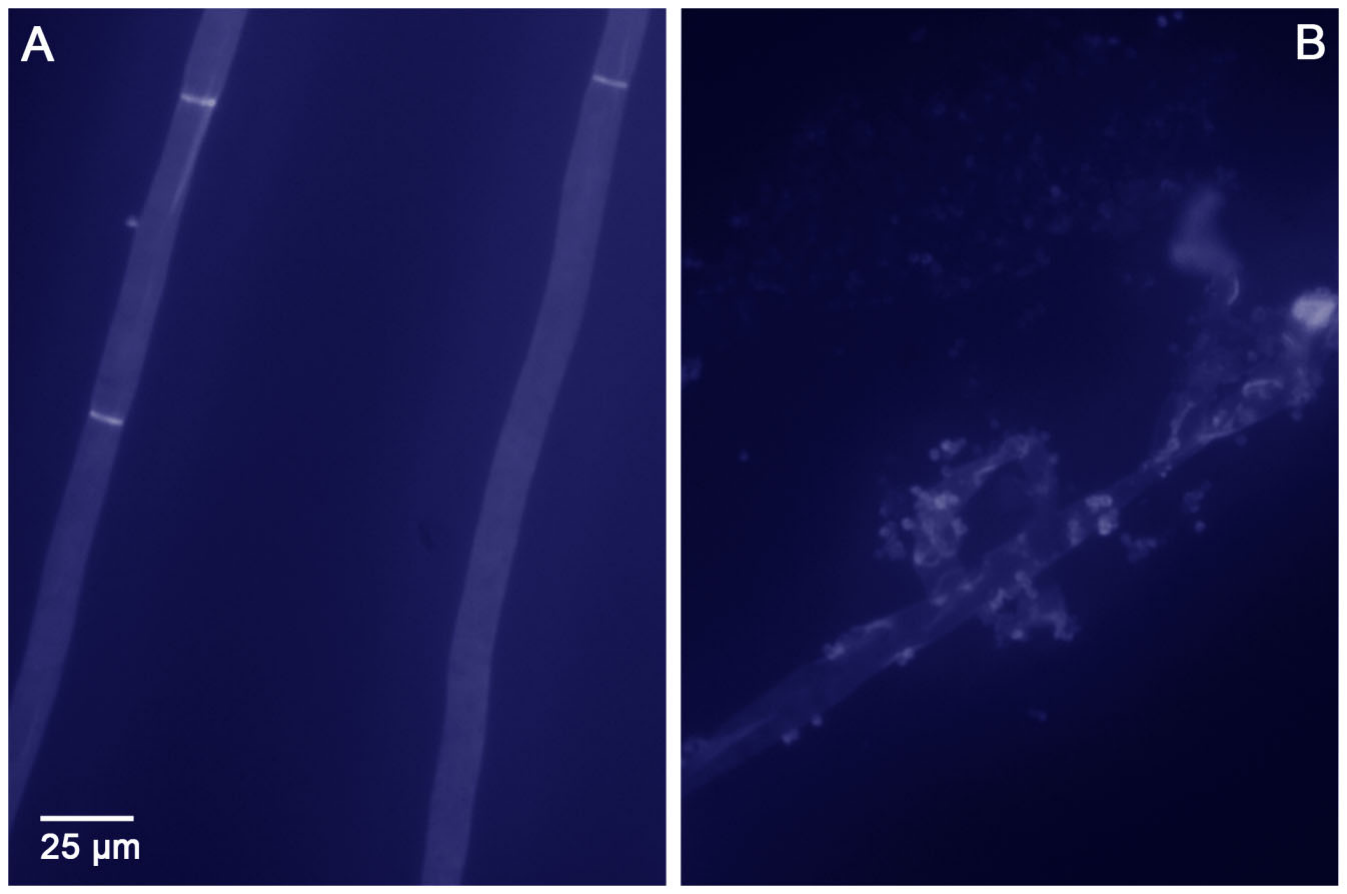
Calcofluor White staining of *in vitro* cultured *A. fumigatus* hyphae after incubation with recombinant chitotriosidase and recombinant AMCase. When hyphae were cultured on Sabauroud's agar (A), the cell wall remained regular and intact after incubation with the two recombinant chitinases. When hyphae were cultured on Sabauroud's agar with 1 mg/L caspofungin (B), the cell wall was irregular and disrupted after incubation with the two recombinant chitinases.

We aimed to confirm this important finding in an alternative experiment. It was not possible to conduct a checkerboard titration with caspofungin and both recombinant chitinases, due to limited availability of recombinant enzymes. Other types of viability assays were unfortunately also not successful. Instead of a viability assay, we then stained the fungal cell wall with Calcofluor White after exposure to caspofungin and both recombinant chitinases. Figure 4A shows unexposed fungal cells and Figure 4B shows caspofungin-exposed cells, both after incubation with a combination of recombinant chitotriosidase and recombinant AMCase. These panels are representative for the complete culture. In other words, most fungal hyphae that were exposed to both chitinases and caspofungin were disrupted. The cells unexposed to caspofungin look very regular with a normal cell wall, with Calcofluor staining mainly seen in regularly spaced septa, whereas the caspofungin-exposed cells show an irregular cell wall with a fragmented aspect. When fungal cells were incubated with a single recombinant chitinase, the cell wall remained regular. When fungal cells were exposed to caspofungin only, the cell wall looked very regular and similar to that in Figure 4A. Thus, in this assay, we confirmed the earlier observations of disruption of the fungal cell wall after exposure to caspofungin and both chitinases. The observed damage to the fungal cell wall makes it very unlikely that the fungal cells remain viable.

## Discussion

The role of mammalian chitinases in the response of invasive fungal infections is a relatively unexplored area of research. In the past it has been shown that chitinase activity was increased in guinea pigs upon *A. fumigatus* systemic infection, but it was not known which chitinase was involved [18], [19]. In the present study we confirmed that chitinases in the lung of immunocompetent rats and of immunocompromised rats were increased after exposure to *A. fumigatus* conidia. We demonstrated that both chitotriosidase and AMCase play a role. We showed that in immunocompetent rats with an appropriate immune system, transient exposure to a high load of conidia without subsequent lung infection resulted in only a moderate increase in the production and activity of chitinases in the lung. Based on our findings, we expect that in certain groups of immunocompetent *A. fumigatus*-colonized patients (such as cystic fibrosis patients), chitinase activity levels would be slightly increased on a permanent basis. We studied chitinase activity in rats with IPA, though extrapolation to the human situation should be done with care.

In all immunocompromised rats developing IPA after inhalation of conidia, chitinases were produced, even though the rats were in neutropenic state. Since *A. fumigatus* itself also produces chitinases, we had to determine if the chitinases measured with our assays were of host origin. We therefore included *in vitro* controls for both the enzyme-assays and immunohistochemistry experiments. These controls were prepared by growing *A. fumigatus in vitro*, without animal cells. No enzyme-activity was observed with the substrates used and no binding of the AMCase and chitotriosidase was observed. Since *in vitro* simulations are not completely representative for the *in vivo* situation, it is not easy to rule out that the fungal chitinases, which might be induced *in vivo* only, were not cross-reacting in our assays. The assays used in our study were similar to the assays used by Overdijk et al [18], [19]. He demonstrated with a Bio-Gel P-100 gel filtration assay that the chitinase activity in the lung consisted of two peaks, one of 35 kDa and one of 15 kDa, which both appeared to be true chitinases [19]. To rule out a fungal origin of the chitinase activity measured in the lungs of *A. fumigatus* infected guinea pigs, Overdijk et al demonstrated that the *A. fumigatus* chitinases eluted much earlier from the column than the mammalian chitinases [19]. Furthermore, allosamidin reduced the chitinase activity in infected and in uninfected guinea pigs by 94% [18]. His final proof was that the activity ratio with the substrates 4-methylumbelliferyl-*N*-acetylglucosamine and 4-methylumbelliferyl-*N*-acetylgalactosamine differed significantly between *A. fumigatus* chitinases and chitinases present in guinea pig serum [18].

It is known that chitotriosidase and AMCase are produced by several cells, such as alveolar macrophages, epithelial cells and neutrophils [11], [22], [23]. Our observations show that chitinase activity was increased significantly during *A. fumigatus* infection, even in rats with decreased numbers of neutrophils and macrophages. During progression to IPA, both the amount of fungal material and the chitinase activity increased in the lung over time.

Treatment with antifungal agents reduces the fungal mass present in the lung. Therefore it was not surprising that Overdijk *et al.* found that treatment of systemic aspergillosis with itraconazole or amphotericin B limited the increase in chitinase activity in guinea pigs [18]. Caspofungin belongs to another class of antifungal agents. It restricts the growth of *A. fumigatus* in the lung and it alters the cell wall composition by decreasing the amount of β-glucan and increasing the amount of chitin in the fungal cell wall [6]. Since caspofungin both reduces the fungal mass in the lung but also increases the relative chitin concentration within this mass, resulting in more substrate for the chitinases, the chitinase activity could either be decreased or increased upon treatment. In our study we found a trend towards a blunting of the chitinase response following treatment with caspofungin, suggesting that the total amount of fungal cells seems to be more important than the composition of the cell wall in the induction of chitinase expression. The total chitinase activity was lower in lungs of caspofungin treated rats than in untreated rats. In contrast, expression of both chitinases seemed to be higher around the fungal focus in caspofungin treated rats. This could be explained by the methods used. Chitinase activity was measured in homogenate of a complete lung, whereas the immunohistochemistry shown displays only the fungal focus. Thus the images shown are not representative for the complete lung, since the fungal focus is located in only a limited segment of the lung. Furthermore it became clear again that caspofungin does not have fungicidal or fungistatic activity. This was supported by the high GM-index of infected, caspofungin-treated rats (Figure 1A). The high GM-index on day 3 could be explained by the detachment of the galactomannan chain from the β-glucan polymeres, caused by caspofungin treatment. Caspofungin is currently in use as salvage therapy for fungal infections. Future studies will be performed to determine if the chitinases also work synergistic with other antifungal agents such as the azoles and polyenes.

Although total chitinase activity in infected rats was not influenced by treatment with caspofungin, the location of the individual chitinases was substantially changed. Upon caspofungin treatment, both AMCase and chitotriosidase seemed to bind to the fungal hyphae, which was not found in untreated infected rats. Furthermore, galactomannan is released from the cell wall resulting in the unmasking of β-glucan and chitin on the *A. fumigatus* cell wall. Unmasking β-glucan has been shown to result in an increased inflammatory response compared to untreated hyphae [24]. The unmasking of the chitin polymers, the target of the chitinases, seems to cause enhanced binding of chitinases to chitin. Possibly, treatment with caspofungin increased the available mammalian chitinase binding domains in the fungus, which might be responsible for the good clinical outcome of treatment with this agent.

It appeared that AMCase was located on the fungal cell wall, while chitotriosidase was located inside the fungal cells. The observed expression patterns suggest that AMCase and chitotriosidase each have a distinct target location and bind to different parts of the fungal cell. It was shown that recombinant AMCase *in vitro* indeed bound the hyphae but that recombinant chitotriosidase was taken up by hyphae only after exposure to both chitinases, thus mimicking the *in vivo* situation where also both chitinases are present. It seemed that recombinant chitotriosidase needed the exochitinase activity of recombinant AMCase in order to be taken up by the fungus. This suggests a synergy between endochitinases and exochitinases. Bolar *et al* also suggested that endochitinases and exochitinases act synergistically in plants [25]. They showed that plants expressing both types of chitinases were less susceptible to *Venturia inaequalis*, the fungal causal agent of apple scab, than plants expressing one of the chitinases [25]. Our observations suggest that such a type of synergy may also be present in mammals.

The synergy between chitotriosidase and AMCase seemed most important when *A. fumigatus* was exposed to caspofungin *in vitro.* When *A. fumigatus* was incubated with caspofungin and both chitotriosidase and AMCase, a direct cell wall degrading effect was noted. No degradation was found when only one of the chitinases was used or when the fungus was not exposed to caspofungin. Also, when the *in vitro* susceptibility assays were performed with each chitinase alone, or when combined with caspofungin, no direct fungicidal effect was observed. Several other authors also investigated activity of chitotriosidase and AMCase, with varying results [11], [26]. Differences in experimental setup could explain the differences found in antifungal activity.

The cell wall degrading properties of both chitinases in combination with exposure to caspofungin could explain why treatment with caspofungin results in decreased mortality in IPA, in spite of the limited fungicidal properties *in vitro*. However, chitotriosidase and AMCase are not expected to be the only type of host response that will be changed upon caspofungin treatment. Since caspofungin alters the composition of the fungal cell wall, it will also alter the pathogen associated molecular patterns (PAMPs) exposed on fungal cells. This alteration in PAMPs could result in a changed production and expression of other signalling molecules, e.g. cytokines and chemokines, contributing to the process of fungal killing. It is expected that killing of fungal cells is achieved by caspofungin treatment in combination with the immune system by using direct and indirect signalling molecules including chitinases. However, the combined action of signalling molecules and mediators with chitinases needs to be elucidated in the future.

## Supporting Information

Table S1 **Enzyme activities of chitotriosidase and AMCase (in a.u.) in rats in several conditions, prior to inoculation and on day 1, day 3 and day 6 after inoculation.** The conditions represented are immunocompetent rats, immunocompromised rats receiving no treatment at all, immunocompromised rats receiving caspofungin treatment from 24 h after infection and immunocompromised rats receiving caspofungin treatment from 72 h after infection.
(XLS)
